# From pilot to a multi-site trial: refining the Early Detection of Deterioration in Elderly Residents (EDDIE +) intervention

**DOI:** 10.1186/s12877-023-04491-z

**Published:** 2023-12-06

**Authors:** Michelle J. Allen, Hannah E. Carter, Elizabeth Cyarto, Claudia Meyer, Trudy Dwyer, Florin Oprescu, Christopher Aitken, Alison Farrington, Carla Shield, Jeffrey Rowland, Xing J. Lee, Nicholas Graves, Lynne Parkinson, Gillian Harvey

**Affiliations:** 1 Australian Centre for Health Services Innovation and Centre for Healthcare Transformation, School of Public Health and Social Work, Faculty of Health, Queensland University of Technology, Brisbane, Australia; 2 School of Public Health and Social Work, Faculty of Health, Queensland University of Technology, Brisbane, Australia; 3 Bolton Clarke Research Institute, Forest Hill, Victoria Australia; 4 Rehabilitation, Ageing and Independent Living Research Centre, Monash University, Melbourne, Australia; 5 Centre for Health Communication and Participation, La Trobe University, Bundoora, Australia; 6 Central Queensland University, Norman Gardens, Australia; 7 School of Health, University of the Sunshine Coast, Sippy Downs, QLD Australia; 8 Faculty of Medicine, University of Queensland, Herston, Australia; 9 Faculty of Health, School of Nursing, Queensland University of Technology, Kelvin Grove, Australia; 10 Metro North Hospital and Health Service, Royal Brisbane and Women’s Hospital, Herston, Australia; 11 Duke-NUS Medical School, Singapore, Singapore; 12 School of Medicine and Public Health, University of Newcastle, Callaghan, Australia; 13 Caring Futures Institute, College of Nursing and Health Sciences, Flinders University, Adelaide, SA Australia

**Keywords:** Residential aged care, Clinical deterioration, Older people, Avoidable hospitalisations, Intervention development, Scale-up, Implementation

## Abstract

**Background:**

Early Detection of Deterioration in Elderly Residents (EDDIE +) is a multi-modal intervention focused on empowering nursing and personal care workers to identify and proactively manage deterioration of residents living in residential aged care (RAC) homes. Building on successful pilot trials conducted between 2014 and 2017, the intervention was refined for implementation in a stepped-wedge cluster randomised trial in 12 RAC homes from March 2021 to May 2022. We report the process used to transition from a small-scale pilot intervention to a multi-site intervention, detailing the intervention to enable future replication.

**Methods:**

The EDDIE + intervention used the integrated Promoting Action on Research Implementation in Health Services (i-PARIHS) framework to guide the intervention development and refinement process. We conducted an environmental scan; multi-level context assessments; convened an intervention working group (IWG) to develop the program logic, conducted a sustainability assessment and deconstructed the intervention components into fixed and adaptable elements; and subsequently refined the intervention for trial.

**Results:**

The original EDDIE pilot intervention included four components: nurse and personal care worker education; decision support tools; diagnostic equipment; and facilitation and clinical support. Deconstructing the intervention into core components and what could be flexibly tailored to context was essential for refining the intervention and informing future implementation across multiple sites. Intervention elements considered unsustainable were updated and refined to enable their scalability. Refinements included: an enhanced educational component with a greater focus on personal care workers and interactive learning; decision support tools that were based on updated evidence; equipment that aligned with recipient needs and available organisational support; and updated facilitation model with local and external facilitation.

**Conclusion:**

By using the i-PARIHS framework in the scale-up process, the EDDIE + intervention was tailored to fit the needs of intended recipients and contexts, enabling flexibility for local adaptation. The process of transitioning from a pilot to larger scale implementation in practice is vastly underreported yet vital for better development and implementation of multi-component interventions across multiple sites. We provide an example using an implementation framework and show it can be advantageous to researchers and health practitioners from pilot stage to refinement, through to larger scale implementation.

**Trial registration:**

The trial was prospectively registered with the Australia New Zealand Clinical Trial Registry (ACTRN12620000507987, registered 23/04/2020).

**Supplementary Information:**

The online version contains supplementary material available at 10.1186/s12877-023-04491-z.

## Background

There has been a 31% increase in admissions in aged care services in the last decade, and today more than 200,000 Australians live in residential aged care (RAC) homes [1]. Previous studies estimate that 5%-60% of transfers from RAC homes to hospital emergency departments are potentially avoidable [2]. Hospitalised residents who are frail often experience increases in mortality, healthcare-associated complications, and resource use even after relatively short admissions (< 72h) [3].

Hospital avoidance initiatives have been developed to promote better resident and health service outcomes. ‘Early Detection of Deterioration In Elderly residents’ or ‘EDDIE’ is a model of care developed and piloted at a not-for-profit aged care organisation in regional Queensland, Australia [4]. Two EDDIE pilots were conducted between 2014 to 2017. They aimed to enhance the ability of RAC home staff to respond appropriately to early signs of resident deterioration. Subsequent evaluation of the pilot interventions showed reductions in the number of hospital bed days used by RAC home residents [4].

EDDIE + is an expanded and refined intervention that has been implemented and evaluated using a stepped-wedge cluster randomised trial in 11 RAC homes across Queensland, from March 2021 to May 2022 [5]. Unlike many hospital avoidance programs, EDDIE + was RAC home driven and promotes practice improvement so that residents who are deteriorating can be identified early and managed proactively. The EDDIE (pilot) and the EDDIE + (trial) intervention comprised of four core components: education and training of all nursing and personal care workers (PCWs); use of decision support tools; use of diagnostic equipment for clinical assessment and monitoring; and facilitation and clinical support [5].

Bridging the divide between pilot research and large-scale implementation is often fraught, resulting in many interventions with promising pilot study evidence, failing to be successfully adopted more broadly [6]. This can occur for multiple reasons such as feasibility of all components at scale and the mediating effects of contextual factors, especially where these are inadequately assessed or adjusted for. To address this challenge, the use of an implementation framework that could guide the study design and account for potential contextual barriers and enablers was considered essential by the research team.

The integrated Promoting Action on Research Implementation in Health Services (i-PARIHS) framework [7] was selected to guide the process of EDDIE + intervention refinement and subsequent implementation. The i-PARIHS framework is a widely used implementation science framework that considers successful implementation to be the achievement of project goals including the uptake and embedding of the intervention (termed the innovation in i-PARIHS), engagement and ownership of the innovation by the intended implementers (recipients in i-PARIHS) and effective tailoring of implementation to context [7, 8]. These aims reflect accepted implementation outcomes such as acceptability, appropriateness, adopting, feasibility, fidelity, and sustainability [9]. The mechanism proposed as the active ingredient for implementation success in i-PARIHS, is facilitation – a role or roles enacted by facilitators who employ enabling facilitation strategies and processes [7, 8].

i-PARIHS was identified as a good fit for the project, as facilitation and clinical support is one of the key components of the EDDIE + intervention, and, given that implementation would be in multiple sites, context needed to be comprehensibly considered. Additional guidance was also sought from the 2008 Medical Research Council’s (MRC) guide ‘Developing and evaluating complex interventions’ [10], due to its focus on operationalisation. The MRC guide (2008 version [10], and updated 2021 version [11]) recommends processes such as interrogation of program logic or theory including what is core (i.e. required as a minimum) and adaptable (i.e. able to be adjusted), consideration of feasibility, inclusion of process evaluation and economic evaluation as part of the research, and consideration of the sustainability and the long-term monitoring of practices.

Of note, four years had passed between the pilot studies and development of the multi-site intervention trial. New evidence and policy changes in the aged care sector meant review and refinement were necessary. Further, the intervention was to be implemented in a stepped-wedge cluster randomised trial, across multiple sites, so a clear structured process for planning and evaluating the implementation was essential for the required rigour of this study design, and to inform the proposed process evaluation [12]. In addition, Australia’s Royal Commission into Aged Care Quality and Safety had released its findings on 1^st^ of March 2021, highlighting the numerous challenges faced by the sector [13]. These contextual developments further enhanced the need for a transparent, considered process for any adaptations to the intervention, and all proposed interactions in and around RAC homes.

A commonly cited criticism in health research is that the reporting of interventions lack sufficient detail to enable future replication [14, 15]. Our aim was to refine the pilot intervention using the i-PARIHS framework and MRC guide to enable the successful implementation and evaluation of the EDDIE + intervention across multiple sites in a sustainable and acceptable way. Within the word limit available, this paper provides information on that process of development and refinement from pilot to trial, as well as a detailed description of the intervention that was subsequently implemented. This approach may serve as an exemplar in the application of a framework to enhance the final implementation, and aid in refining an intervention to align to the complex and challenging environment of a large multi-site trial. By documenting and reporting this information, we hope to aid scholars, practitioners and policy makers navigating this complex and often opaque part of implementation research.

## Methods

### Design and alignment of methods to framework

Applying i-PARIHS, the process of reviewing and refining EDDIE + was informed by the constructs of innovation, recipients, context (inner and outer) and facilitation using the following methods:

1. Environmental scan (outer context)
2. Organisational and local RAC home context assessments (inner context and recipients)
3. Intervention working group (innovation, facilitation, recipients)

Outputs from the aforementioned, informed subsequent intervention refinements (innovation, recipients, and facilitation) related to barriers and facilitators to implementation, including development of updated educational resources, decision support tools, and materials to support local on-site clinical facilitators.

### Environmental scan (outer context)

An environmental scan was conducted as a desktop review by the project team to assess the broader health care context, defined as the ‘outer context’ within the i-PARIHS framework. This included a review of relevant programs and policies nationally, and evidence relating to hospital avoidance and the aged care sector in Queensland. Multiple websites of peak bodies, private providers and government departments were searched including Council on the Ageing, Aged and Community Services Australia, Australian Association of Gerontology, Royal Australian College of General practitioners, Department of Health and Aged Care, and the Royal Commission into Aged Care Quality and Safety.

Key search terms included hospital avoidance, residential aged care, aged care, dementia, and frailty. A full list of organisations and associated search strategy can be found in Additional file 1. Information on current programs related to hospital avoidance for each Queensland Hospital and Health Service or Primary Health Network area (as defined in [16]) were collated in a spreadsheet, noting potential impact or interaction with the EDDIE + intervention. Resources and other key documents such as program factsheets, strategy documents, and clinical guides were also collated and reviewed.

### Context assessments (inner context and recipients)

Context assessment templates were developed by the study team based on the i-PARIHS framework to map the inner context (both the organisational and individual/local RAC home levels) and information on the EDDIE + recipients, defined as the RAC staff. The organisational context assessment collected information on the organisational structure (including governance, reporting, and staffing), number and size of RAC homes, key policies, and procedures, as well as organisation wide professional development programs (Additional file 2). The organisational level context assessment was carried out virtually by the nurse educator (EDDIE + study team) and key clinical executives from the aged care provider between December 2020 and January 2021.

Local RAC home level context assessment templates were developed for use during the trial to gather information on specific RAC home characteristics such as location, number of beds, local processes for staffing and reporting, policies, and procedures for interacting with general practice, allied health and the local hospital, and available hospital or health service support on deterioration, then align that information to implementation priorities (Additional files 3 and 4). The local context assessments were conducted in 11 RAC homes in Queensland, Australia, from March 2021 to May 2022, as part of the stepped-wedge cluster randomised trial of the EDDIE + intervention.

As the aged care provider is a national not-for-profit organisation with multiple facilities, a mix of metropolitan and regional sites across Queensland, Australia, were purposively selected for inclusion in the trial. RAC homes ranged in size from 91 -164 bed homes, staffed by a workforce of 53–81 Personal Care Workers (PCWs) and 12–28 Registered Nurses (RNs) and Enrolled Nurses (ENs). More details on the trial design can be found in the published protocol [5]. The intervention development phase described in the current paper was undertaken from mid-2019 to March 2021.

These assessments were conducted by EDDIE + study team members (nurse educator and implementation facilitator) with the local EDDIE + clinical facilitator and management team (e.g. residential manager, clinical manager) of each RAC home. Local RAC home context assessments were conducted during the first meeting with each home, as part of the pre-trial establishment activities, in order to tailor any adaptable elements of EDDIE + to their context and recipients. The establishment phase occurred at different times for each home, as per the stepped-wedge study design. Outcomes of these meetings were discussed within the project team, as to whether any adaptations were expected or needed to tailor to each RAC home.

### Intervention working group (innovation, recipients and facilitation)

An Intervention Working Group (IWG) was convened with an initial focus on reviewing the EDDIE pilot intervention and identifying requirements for scaling up to multi-site implementation. The goal was to ensure the feasibility and scalability of the project. There was an expectation that a level of adaptation would be required for the participating RAC homes, given the mix of metropolitan and regional locations, varied home sizes, and the different populations served. Further, shifting from small scale pilot studies in single sites to implementation across multiple sites, with a different aged care provider from the pilot phase required consideration.

The initial convening of the IWG included investigators involved in the pilot intervention, those from the participating aged care provider, and investigators able to provide implementation science expertise and clinical expertise, covering a range of perspectives. The university-based study team responsible for implementing the trial were also part of the IWG. See Table 1 for IWG participant information. The working group met four times virtually between December 2019 and January 2021. The working group agenda considered findings from the environmental scan, organisational context assessment, lessons learnt and the outcomes of the pilot evaluation, and any changes in evidence or guidelines since the pilot.

**Table 1:** Intervention working group participants

Role within Project	Specialist area	Gender	Involved in pilot study (Y/N)
Chief Investigator	Implementation science, Nursing, Aged Care	F	N
Chief Investigator	Nursing, Aged Care	F	Y
Chief Investigator	Health economics	F	Y
Chief Investigator	Nursing, Aged Care	F	N
Chief Investigator	Gerontology, General medicine	M	N
External	Nursing, Aged Care	F	Y
Project Manager	Project Management, Nursing	F	N
Project Coordinator	Project Management	F	N
Nurse Educator	Nursing, Education	M	N
Implementation Facilitator	Implementation science	F	N

The MRC guide states that a key step in scaling up complex interventions is the “deconstruction” of the components of an intervention, and an agreement on what variation is acceptable and what variation is prohibited [10, 11]. Consensus was achieved through consideration of different perspectives and a focus on pragmatic and sustainable implementation. This level of detail was necessary to inform the local tailored implementation at each RAC home, and to guide the evaluation of fidelity within the process evaluation. Each tool and process used in the pilot intervention was also assessed for feasibility and sustainability at scale, including whether intervention components could be appropriately staffed or resourced, the expected costs within and beyond the trial phase, their use across contexts, and their suitability for a trial environment. All IWG members reviewed the evidence for each criteria, and decisions were made by consensus (min 75% IWG member agreement).

All intervention refinements (from pilot to trial protocol) were led by the study team, with input from the aged care provider and reviewed by the IWG against the program logic (Additional file 5) to ensure the intent and mechanisms of change were maintained or enhanced. During the trial, if local requirements needed an adaptation, and it had already been defined that the adaptation was suitable for a particular aspect, review by the IWG was not required beforehand, but was conducted as part of governance and fidelity.

## Results

### Environmental scan (outer context)

The environmental scan identified policies, plans and strategies of peak bodies, research grant calls, and the Royal Commission into Aged Care Quality and Safety from the broader environment that could impact the way EDDIE + was received or even delivered. For example, the Royal Commission’s recommendations in relation to improvements to workforce conditions and capacity had the potential to boost the acceptance of EDDIE + due to alignment with better training for carers, but equally EDDIE + might be seen as an additional strain if RAC homes already had additional training that needed to be delivered during the trial period.

The environmental scan also revealed recent or current hospital avoidance programs and initiatives, training resources, and relevant policies, plans and strategies. Several existing hospital avoidance programs and initiatives were identified. These programs were implemented at various locations and facilitated by different providers such as local Hospital and Health Services, Primary Health Networks, health service providers or university groups. Recently concluded programs were also noted, with several leading to reduction in hospital usage. No other hospital avoidance programs were targeted towards inclusion of PCWs, and all were “in-reach” hospital programs not RAC home led initiatives. These programs were considered potential enablers in terms of access to information or support for RAC staff. Hospital avoidance program components, geographical reach, and potential impacts on RAC homes were identified. Where RAC homes were identified as having access to one or more of the existing hospital avoidance programs, awareness for staff of these programs was incorporated into training and decision support processes for nursing staff.

### Context assessments (inner context and recipients)

Key findings from the organisational context assessment included capacity and capability enablers such as strong clinical governance structures; the availability of Senior Clinical Nurse Advisors (SCNAs) for key specialties within the aged care provider (including continence, dementia, and palliative care); availability of an integrated system for all residents’ information; and availability of a learning management system which already included modules on deterioration. There was no evidence that trial RAC homes had bladder scanners or the proposed vital sign monitors onsite at that time, making the EDDIE + intervention potentially attractive to RAC home staff. This organisational level context assessment also recognised barriers such as information transfer between RAC homes and the hospital system, and long-term systemic issues within the broader aged care sector around staff recruitment and retention. These findings further emphasised the need for the local level facilitation, and connection between nurses and PCWs and internal support structures such as the SCNAs. Results from local RAC home level context assessments were undertaken during the trial phase (not during development) so will be reported elsewhere.

### Intervention working group (innovation, recipients, facilitation)

A key finding from the pilot evaluation was the importance of all four intervention components working synergistically. The IWG agreed these components were core and necessary for the trial phase, but all required refinement. A table with each of the core components of the intervention broken down into elements, including the extent of variation permissible for each element (fixed, moderate, extensive), was developed by the study team, and reviewed by the IWG (as per Additional file 6) before progressing.

#### Refinement of training

The educational component of the pilot intervention targeted both nursing staff (RNs and ENs) and PCWs. Results from the environmental scan demonstrated a gap in training and resource material specifically for PCWs. Thus, it was agreed by the IWG that a specific set of training materials was needed for PCWs to better match their role and needs. Two distinct sets of training materials were then developed (by the study team led by the nurse educator), to better align with each professional group’s needs. The training content refinement process included a literature search; a review of the Aged Care standards and Queensland Health policies; a review of the aged care provider’s training modules, policies, and procedures; observation of current training sessions and online modules to become familiar with the online learning platform currently in use; and consultation with key personnel at the aged care provider.

There were eight areas of deterioration identified as commonly leading to potentially avoidable hospitalisations in the pilot evaluation (delirium, constipation, dehydration, dyspnoea, cardiac, urinary tract infections, falls and palliative care), and it was agreed to continue with these as the focus areas of the education and training component and decision support tools in the larger trial.

In addition, a barrier cited in previous research was the communication barrier between PCWs and nursing staff when reporting deterioration [17]. As such, content was added to the training and decision support tools to focus on the communication mechanisms. Communication-specific refinements included:

- Introduction of the two communication tools, “Stop and Watch” and “CUS” for PCWs to report deterioration to nursing staff. Implementation of the “Stop and Watch” communication tool was planned for 2021 by the aged care provider, aligning with licensing requirements. This tool uses the phrase “Stop and Watch” to draw attention to key aspects of deterioration. The “CUS” communication tool uses the phrases “I am Concerned…”, “I am Uncomfortable”, and “This is a Serious/Safety issue” to focus the nurses’ attention [18].
- Expectation for nursing staff to ‘close the loop’ by providing feedback to PCWs on the actions taken by the clinical team after deterioration had been reported.
- Wearable reference cards with communication tool wording provided to PCWs in response to initial feedback that identified a need to provide point-of-care reference materials to aid PCWs and nursing staff in their communication.
- Nursing staff provided with notepads branded with communication and feedback loop reminders.
- Communication-specific scenarios developed to support staff reinforcement of reporting and feedback practices.

These additional communication-specific tools were assessed to be in line with the mechanisms of change that were in the original program logic. Further, the educational content was reviewed with an adult learning and communication lens to maximise knowledge transfer and information retention by both clinical and non-clinical recipients, and to support virtual or hybrid delivery during COVID-19. Enhancements included:

- Introductory 1-2min video for all staff on the purpose of EDDIE + 
- Development of a four-step process of Recognition, Assessments, Care, and Referral/Review (RACR) to structure the information in small, repeated sections within each of the eight areas of deterioration
- Repeated visual cues with coloured hexagon icons for each of the eight areas of deterioration for both PCW and nursing staff
- Use of non-clinical language with PCWs focusing on symptom recognition and other signs of deterioration
- Clinical scenarios for use by the local EDDIE + Clinical Facilitator in each site to support ongoing detection of deterioration throughout the trial phase (as a form of reinforcement of learnings)
- A specific procedure for the administration of subcutaneous fluid was incorporated into training after the RAC home provider identified the requirement to re-introduce the procedure in preparation for possible changes to hospital practices during COVID-19 outbreaks.

#### Decision support tool refinement

Assessment of the feasibility and sustainability of the original EDDIE intervention elements identified intellectual property restrictions, licencing considerations and cost requirements for some elements of the pilot including innovative teaching simulation techniques such as “Mask-Ed” and the “traffic light" deteriorating resident decision-making tool. For example, Mask-Ed ™(KRS Simulation) uses realistic silicone masks and body suits to create a platform for realistic teaching and learning [19]. The Residential Acute Deterioration Detection Index (RADD or traffic light system) [20] is a decision-support tool that used the “traffic light” colour system to assist nursing staff to detect, refer and quickly respond to early signs of a deteriorating resident.

The aforementioned limitations were not considered conducive to the sustainability and scalability of the intervention; therefore, they were not included in the larger study and suitable replacements were sourced or developed. This included multiple point-of-care formats to enhance accessibility of information, exposure to additional assessment tools (the CAM—Confusion Assessment Method [21], Borg Dyspnoea scale [22], PAINAD—Pain assessment for advanced dementia [23], and SPICT™—Supportive and Palliative Care Indicators Tool [24]), and alignment with the latest clinical parameters outlined in *‘The Management of acute care needs of RACF residents—A suite of collaborative pathways for General Practitioners and Registered Nurse, Queensland Health, 2019 (Version 21)*’, which had been published after the pilot studies [25]. These additional assessment tools were not considered a core component of the EDDIE + intervention but were added to the suite of available materials for nursing staff after consultation with the Aged Care provider.

#### Diagnostic equipment

A key component of the EDDIE + project was the procurement and installation of clinical equipment to assist with early deterioration and care management of residents. A comparative chart of features, benefits, capacity, quality, consumables, and costings was collated to ascertain the most suitable equipment. This information was reviewed by the study team and key personnel from the aged care provider including the SCNAs. Initially, a 12-lead Electrocardiogram (ECG) as part of the vital signs monitor was considered. However, this would require specialist training to use and interpret results, which was decided to be out of scope. Instead, a vital signs monitor with three lead ECG, to assess rhythms, and store and print out key data for monitoring and reporting to doctors or the hospital team, was agreed to be a more feasible option. Multiple bladder scanners were reviewed. In consultation with the aged care provider’s SCNA for continence it was decided that using a make and model currently used by the participating aged care provider in another Australian state, would provide the continuity and standardisation of support needed. This support included access to the SCNA, which was an important aspect of the sustainability of EDDIE + beyond the trial. Hands on simulation training (with a mannequin and phantom bladder), was included for all nursing staff on the provided equipment.

#### Facilitation model changes

The local home-based Clinical Facilitator was noted to be a vital element of the implementation process in the pilot. In line with the i-PARIHS framework, it was agreed that this role was an essential component of EDDIE + . Appropriate resourcing was also considered important to enable the local EDDIE + Clinical Facilitator to enact the role, particularly given the organisational and broader context of aged care. Consequently, funding was provided for a 0.2 full time equivalent (FTE) EDDIE + Clinical Facilitator per site during the study (facilitator role not funded in the pilot).

To accommodate shift requirements and individual RAC home demands, the option of sharing of the EDDIE + Clinical Facilitator role between more than one nurse was included. It was also agreed that the local EDDIE + Clinical Facilitator would benefit from additional support and mentoring from a more experienced facilitator, which aligns with the i-PARIHS framework and the idea of different levels of facilitation. As such, external facilitation via the study implementation facilitator and nurse educator roles (both from the university-based study team) would support implementation across the sites, which was not part of the original pilot.

Communication with residents, families, general practitioners, and other stakeholders was highlighted in the pilot evaluation to be a vital element needed to create the type of buy-in that supports successful implementation. However, direct interaction with residents and their families for study purposes was limited by the aged care provider, due to the increased sensitives and pressures in the aged care sector, and sustained COVID-19 pandemic priorities. Further, unlike the active engagement of general practitioners and local hospitals in the pilot studies this engagement was minimal during the trial phase.

### Description of final intervention (as delivered)

The final EDDIE + intervention is described in detail in Table 2, including who undertook which activities, when, and the materials used in each instance, as per the TIDieR reporting guidelines [14]. This is additional information not previously reported in the initial protocol paper [5]. A summary of what changed from the pilot to the trial intervention can be found in Additional file 7.

**Table 2:** Final intervention details

What	When	Materials	How delivered and by whom	Data collected
PCW introductory education	Within first 4 weeks^a^ of intervention introduction phase	• Introductory video – What is EDDIE + ? • PowerPoint slides covering: – What to look for – signs and symptoms of early deterioration (eight areas) – What to say (communication with nursing staff) via communication tools CUS & Stop and Watch • Workbook • EDDIE + pen and chocolate	Nurse educator presents key information in a blended learning style with question-and-answer (1 h session) Face-to-face or online option – conducted in groups Each participant received once	• Attendance records • Presenter feedback form • Self-efficacy questionnaire^b^
Nurse introductory education	Within first 4 weeks^a^ of intervention introduction phase	• Introductory video – What is EDDIE + ? • PowerPoint slides covering: – Eight areas of clinical deterioration – RACR – Recognise, Assess, Care, Report (see other section for more detail) – Communication to GPs and other clinical services e.g. ISBAR tool – Communication with personal care workers covering the CUS & Stop and Watch tools they will use for reporting, and expectation for closing the feedback loop with PCWs • Workbook • EDDIE + pen and chocolate	Nurse educator presents key information in a blended learning style with question-and-answer (1h 15min session) Face-to-face or online option – conducted in groups Each participant received once	• Attendance records • Presenter feedback form • Self-efficacy questionnaire^b^
Equipment training	Within first 4 weeks^a^ of intervention introduction phase	• Each site was provided with two pieces of equipment—(1) Bladder scanner; and (2) Vital signs monitor with blood pressure, pulse oximetry, and 3 lead ECG; with alarms, automated observation cycling (e.g. every 15 min), and print-out options • 2–3 PowerPoint slides on interpretation of results and management of clinical findings • Phantom bladder, distilled water, ECG gel • ANNIE demonstration mannequin, ECG dots, vital signs monitor observation print-out strip • Workbook and competency quiz (in addition to hands-on assessment) • Instructional videos available to staff for revision • Quick reference guides attached to each piece of equipment for future referral	Nurse educator presents 2–3 slides, conducts a hands-on demonstration with both pieces of equipment, including use of phantom bladder. A hands-on competency assessment, as well as short quiz is completed by nursing staff (45 min session in total for both pieces of equipment) EDDIE + Clinical facilitator in each site provides additional support or revision training for nursing staff in using equipment as needed	• Attendance records
Bladder Scanner	From start of intervention introduction phase to end of intervention exposure phase (available for use once trained)	• 1 × bladder scanner per site • Consumables e.g. ultrasound gel	Provided to each site at the beginning of the intervention introduction phase (with consumables provided throughout) Nursing staff to use with residents as needed throughout the intervention introduction and exposure phases (once training complete and assessed as competent)	• Fortnightly CF check-in form
Vital signs monitor	From start of intervention introduction phase to end of intervention exposure phase (available for use once trained)	• 1 × vital signs monitor per site • Consumables e.g. ECG dots and paper	Provided to each site at the beginning of the intervention introduction phase (with consumables provided throughout) Nursing staff to use with residents as needed throughout the intervention introduction and exposure phases (once training complete and assessed as competent)	• Fortnightly CF check-in form
Decision Support tool	Within first 4 weeks^a^ of intervention introduction phase	• Retractable badge holder with key decision support/ information attached (2 double sided cards for nurses, 2 double-sized cards for PCW) • Laminated copies of key assessment tools that are kept in each nurses’ station	Provided during introductory education session for PCWs and Nurses. Staff to wear throughout trial (to end of intervention exposure phase)	• Fortnightly CF check-in form
Individual RAC home context assessment (baseline)	Initial site visit 3–4 weeks prior to intervention introduction phase	• Individual RAC home context assessment based on the i-PARIHS framework	Implementation Facilitator 1 h session (usually F2F) with local Residential Manager, Clinical Manager and Clinical Facilitator	• Individual RAC home context assessment • Field Notes
Clinical Facilitator induction training and materials	Initial site visit 3–4 weeks prior to intervention introduction phase	• Clinical facilitator guide (printed and electronic versions) • USB and printed versions of all project materials including: – Promotional posters – Data collection tools such as check-in form, training logs – Training materials – Engagement letter/information for key stakeholders • Laptop bag with additional tools for the facilitator e.g. diary, notepad, pens, BlueTak	2–3 h session with EDDIE + Clinical Facilitator at each site, Nurse Educator and Intervention Facilitator Face-to-face or online option	• Field notes
Onsite EDDIE + Clinical Facilitator	From initial site visit (3–4 weeks prior to intervention introduction phase) through to end of intervention exposure phase	• Each site had 0.2FTE nursing position paid for by the study	Clinical facilitator to: • Support implementation of the EDDIE + program, including training and mentoring activities, maintaining nursing and care staff and RAC home engagement • Support and monitor nursing and care staff use of the EDDIE + processes, equipment, and decision support tools in the home • Speak with the Nurse Educator or Implementation Facilitator fortnightly to maintain RAC home engagement, monitor implementation processes and address issues as required • Documentation and record keeping of, staff training, consumable usage and equipment maintenance using the template/s provided • Liaison with study team to provide data • Work with the Nurse Educator to identify and engage with clinical support channels as required, including engagement with GP practices, nurse practitioners • Assist with resident and family/ advocate/ representative engagement with the EDDIE + program • Advocate for and champion the EDDIE + Project in the RAC home to all nursing and care staff, residents and families • Work with the Nurse Educator to identify local and individual learning needs • Undertake extension scenarios with staff (see detail below) • Assist with the local orientation of all new staff to EDDIE +	• Fortnightly CF check-in form
Extension scenarios	Throughout the implementation exposure phase	• Clinical and communication scenarios with 2–3 questions per scenario– available electronically on a tablet and hard copy with a verbal discussion of the questions • Android tablet with data (included videos and scenarios)	Clinical facilitator in each site undertakes scenario discussion on a semi-regular basis with nurses and PCW (5min to 30min sessions during shift “on the floor”)	• Fortnightly CF check-in form
Mentoring and support for Clinical Facilitators	From initial site visit (3–4 weeks prior to intervention introduction phase) through to end of intervention exposure	• Fortnightly check-in meetings between Clinical Facilitator and EDDIE + Nurse Educator (or EDDIE + Facilitator) • Availability of coaching on teaching techniques • Availability of list of contact information for RAC home clinical nurse advisors	Nurse Educator and Implementation Facilitator regularly engaged with each EDDIE + Clinical Facilitator by email and phone, and any activities as required EDDIE + Clinical Facilitators were encouraged to access internal BC supports such as SCNAs and other Clinical Facilitators at participating sites	• Individual RAC home context assessment • Fortnightly CF check-in form • Field Notes

^a^Can occur at later stage for staff that were either unavailable or had not started employment when initial training was first delivered

^b^Self-efficacy questionnaires were conducted at the beginning of the introductory education session, and repeated at the end of the intervention exposure phase

### Education and training component

#### Introductory training

An introductory training session was designed for delivery by the EDDIE + nurse educator either face to face or online. Introductory training for PCWs covered the signs and symptoms of each of the eight areas of deterioration, along with communication tools to support the reporting of potential deterioration. Introductory training for nurses covered more in-depth clinical assessment and care options, as well as communication tools for them to report to other clinicians and close the feedback loop with PCWs.

#### Equipment training

Equipment training was designed to either run concurrently with the RN/EN introductory training or as an independent session. Only small groups (1–4) attended per session to enable hands on simulation and assessment of competency. A quiz was devised with five multiple-choice questions each for the bladder scanner and the vital signs monitor respectively, which formed part of the competency assessment.

#### Ongoing scenario-based training

Ongoing training was designed to be undertaken in one-on-one or in small groups “on the floor” during shifts, facilitated by the local EDDIE + Clinical Facilitator. Clinical scenarios with associated questions were used to prompt discussion about identifying deterioration, communication, and feedback.

### Decision support and clinical assessment tools

#### Point of care clinical parameters tool for nurses

A clinical parameters tool, based on recent guidance [25], was provided to the nursing staff as a poster and formatted into a clinical decision support card (size of an identification tag) with a clip to attach to their uniform for point-of-care access.

#### Point of care information for PCWs

A decision support card (the size of an identification tag) was created for PCWs with a clip to attach to their uniform for point-of-care access. The information included the eight areas of deterioration, a reminder to notice and report early changes in residents, and the communication tools for reporting deterioration used in the training.

#### Additional decision support/assessment tools

Printed and laminated copies of all assessment tools were provided for each nurse’s station.

### Diagnostic equipment

One bladder scanner and one vital sign monitor were provided to each participating RAC home along with all consumables. All nursing staff were trained (as per the education section) to use the equipment. Instructional videos and double-sided A4 quick reference guides (attached to the equipment) were also provided, and available for use in reinforcement training conducted by the local EDDIE + Clinical Facilitator during the trial.

### Facilitation and clinical systems support

Introduction of a combined internal–external facilitation model required the development of materials to support the local EDDIE + Clinical Facilitator in each site including a facilitator’s guide, orientation training by the study’s implementation facilitator and ongoing support mechanisms. The local EDDIE + Clinical Facilitator role had four key components—staff mentoring and support; ongoing training (scenarios and equipment); conduit with local stakeholders (e.g. doctors, allied health) about the trial; and data collection (see Table 2 for more detail).

Each local Clinical Facilitator attended an orientation and training session with the study implementation facilitator at their RAC home prior to starting the intervention. The Clinical Facilitator guide covered aspects of being an effective facilitator, available clinical support networks, details on the data collection and reporting processes, tips on keeping staff engaged in the program, and tips on engaging stakeholders. A training resource folder with all training materials including lesson plans, scenarios, and other information on the training was provided.

Templates for data collection and communication were also included (e.g. posters, fortnightly check-in), as were a range of resources and materials for their use during the study e.g. trial bag with a tablet & USB (with electronic versions of all key documentation and resources including videos), diary, notepad, and stationery. The guide and other materials were provided to each home during their orientation meeting. Ongoing support and coaching were available to each of the EDDIE + Clinical Facilitators via the study team (specifically the nurse educator and the implementation facilitator) who maintained regular phone, email and visit contact (minimum every 2 weeks), and internally via the SCNAs at the aged care provider.

## Discussion

This work takes the important step of scaling evidence from pilot programs through to a multisite intervention, drawing on implementation science theory through use of i-PARIHS framework and the MRC guide for complex interventions. Steps like the intervention deconstruction process (into core and adaptable elements), assessment of feasibility and sustainability, and the development of detailed context assessment tools are key to the process of scale-up, and the subsequent ability to tailor the intervention to each RAC home without compromising fidelity. Our inclusion of these steps are supported by the most recent version of the MRC guide (2021)—published during our implementation of the EDDIE + trial but after the development phase—highlighting economic feasibility and understanding the process of change as key to maximising the impact of complex interventions [11]. Whilst the importance of tailoring an intervention in aged care has long been supported by research, whether it be tailoring to address determinants of professional practice [26], dementia risk reduction [27], or antimicrobial stewardship in nursing homes [28], detailed description of what is adaptable and what is core is rarely published, so the reader cannot assess whether the adaptations were within planned parameters.

There has been some recent discussion on the limited applicability of theories, models or frameworks in practice and seemingly incongruent nature of implementation science theory in pragmatic trials [29–31]. Rapport and colleagues (2022) highlight the need to operationalise theory in a practical way, as well as integrate implementation scientists with those who are delivering interventions [30]. This paper provides a practical example of how using an implementation framework, can be advantageous to researchers and care providers when transitioning from pilot stage through to larger scale implementation, and how implementation science can be operationalised for a trial setting.

Detail around the final intervention is further described in the Supplementary Files (Files 6 and 7), but some important changes from pilot to trial include multi-layered facilitation and refreshing the training for more interactive delivery, a greater focus on communication, and new evidence informed clinical pathways. The potential impact of these changes, combined with the contextual information on the implementation of the trial will be explored in the process evaluation and trial results papers.

We recognize that the development and refinement of the intervention could have been further enhanced and contextualized by including aged care residents and their families, or local clinicians (e.g. general practitioners) in the IWG or through local resident committees, but was not supported by the aged care provider at the time. Further, the EDDIE + trial was developed in a single Australian state and may require future refinement and adjustment to context if the intervention is to be scaled nationally or internationally.

## Conclusion

This paper describes the EDDIE + intervention in sufficient detail to be replicated. The refinement process from pilot to implementation scale-up including adaptations to context during the process were discussed. The process described may assist other researchers planning and evaluating the scale up of a complex interventions into practice. The combination of an implementation framework (i-PARIHS) and published guidance on developing and evaluating complex interventions was practical and beneficial in this implementation study. These processes are vastly underreported, yet vital for future research in this area.

### Supplementary Information

**Additional file 1. **Environmental scan – Search Strategy. This is the search strategy used when conducting the environmental scan. It includes the websites searched and the terms used.

**Additional file 2. **Organisational context assessment template. This is the template used by the study team when undertaking the organisational level context assessment. The i-PARIHS framework was used in its development to assess the key domains.

**Additional file 3. **Local RAC Home template. This is the template used by the study team to collect key information about each individual residential aged care home.

**Additional file 4. **Local RAC Home context template. This is the template used by the study team to assess barriers and facilitators. This i-PARIHS framework was used in its development covering the key domains.

**Additional file 5. **EDDIE+ Intervention Logic model. This is the program logic model used for the EDDIE+ trial.

**Additional file 6. **Fixed and flexible components and degree of flexibility allowed. This table details the fixed and adaptable components of the EDDIE+ intervention and the degree of flexibility that is allowed during the trial phase.

**Additional file 7. **Changes between pilot intervention and EDDIE+ intervention. This is a table mapping out what was changed between the pilot phase and the final EDDIE+ intervention including the methods and findings of each step.

**Additional file 8. **TIDieR (Template for Intervention Description and Replication) Checklist. This is a completed checklist ensuring the detailed description of the EDDIE+ intervention was included in this paper.

## Data Availability

Data generated or analysed during this study are included in this published article (and additional files) or are available on request from the corresponding author. Additional materials such as the educational resources are freely available on https://www.aushsi.org.au/research/projects/eddie-resources/ for use under a creative commons license.

## Abbreviations

CAM: Confusion Assessment Method
COVID-19: Coronavirus pandemic
CUS: Name of communication tool using the terms – Concerned, Uncomfortable, Serious/ Safety issue
ECG: Electrocardiogram
EDDIE: Early Detection of Deterioration in Elderly Residents (pilot intervention)
EDDIE +: Early Detection of Deterioration in Elderly Residents + (final intervention for stepped-wedge trial)
EN: Enrolled Nurse
i-PARIHS: Integrated Promoting Action on Research Implementation in Health Services
MRC: Medical Research Council
PAINAD: Pain assessment for advanced dementia
PCW: Personal Care Worker
RAC: Residential Aged Care
RACR: Recognition, Assessments, Care, and Referral/ Review process
RN: Registered Nurse
SCNA: Senior Clinical Nurse Advisor
SPICT^TM^: Supportive and Palliative Care Indicators Tool
TIDieR: Template for Intervention Description and Replication Checklist
UK: United Kingdom
USB: Universal Serial Bus
