# Supplementary material for: From pilot to a multi-site trial: refining the Early Detection of Deterioration in Elderly Residents (EDDIE +) intervention
Source: BMC Geriatr. 2023 Dec 6;23:811. doi: 10.1186/s12877-023-04491-z (PMC10698876; doi:10.1186/s12877-023-04491-z)
Supplement: Supplementary file 1 — Additional file 1. Environmental scan – Search Strategy. This is the search strategy used when conducting the environmental scan. It includes the websites searched and the terms used. [file 12877_2023_4491_MOESM1_ESM.docx]

**Environmental Scan search strategy – hospital avoidance, RACF and aged care**

**Sites reviewed:**

ACSA (Aged and Community Services Australia)

LASA (Leading Age Services Australia)

CEQ

PHNs

RACGP

APNA

AAG (Australian Association of Gerontology)

ANZSGM

HICQ/CHF (Consumers Health Forum)

COTA (Council on the Ageing)

HESTA

QNU (Queensland Nursing and Midwifery Union)

QAS

BDHP

APHA (Australian Private Hospitals Association)

AHHA/Deeble

MRFF/NHMRC

HHS

AIHW

Centre for research on ageing, health and wellbeing

ACHS

Bolton Clarke

ACSQHC

Private insurers

National Institute for Dementia Research

DCRC (Dementia Centre for Research Collaboration)

Dementia Training Australia

Dementia Australia/National Dementia Network

DORA

Department of Health

Royal Commission Aged Care

**Search terms within each stakeholder site:**

Hospital avoidance program

Residential aged care facilities

Aged care

Dementia

Frailty
