# Supplementary material for: From pilot to a multi-site trial: refining the Early Detection of Deterioration in Elderly Residents (EDDIE +) intervention
Source: BMC Geriatr. 2023 Dec 6;23:811. doi: 10.1186/s12877-023-04491-z (PMC10698876; doi:10.1186/s12877-023-04491-z)
Supplement: Supplementary file 2 — Additional file 2. Organisational context assessment template. This is the template used by the study team when undertaking the organisational level context assessment. The i-PARIHS framework was used in its development to assess the key domains. [file 12877_2023_4491_MOESM2_ESM.docx]

**BASELINE organisational information**

**Date: Completed by:**

Purpose:

- To systematically collect information from Bolton Clarke to inform local tailoring of all the EDDIE+ activities, including training development and delivery, engagement with staff, resident committees and local primary care providers

| **Information required: ORGANISATION NAME**  ***(focus on core, organisational-wide practices)*** | **Source** | **Notes** |
| --- | --- | --- |
| **ORGANISATIONAL STRUCTURE & CHARACTERISTICS** | | |
| Number of homes |  |  |
| Numbers of beds (current and capacity) |  |  |
| Accreditation |  |  |
| Committee/reporting structure |  |  |
| Executive support and engagement |  |  |
| **PEOPLE: Staff** | | |
| Organisational structure |  |  |
| Nurse and care staff position descriptions / duty statements, awards/wage ranges |  |  |
| Key positions for EDDIE+ |  |  |
| Staff training and education schedule and content |  |  |
| New staff induction schedule and content |  |  |
| Meeting structure (nursing and care staff, homes, management) |  |  |
| Communication mechanisms |  |  |
| Backfill arrangements |  |  |
| **PEOPLE: Residents, family and nominated advocates** | | |
| Resident communication mechanisms and frequency |  |  |
| Family and nominated advocate communication mechanisms and frequency |  |  |
| **STAKEHOLDERS:** *See Project Plan – 8. Stakeholder analysis* | | |
| **KEY POLICIES & PROCEDURES** | | |
| Location and access |  |  |
| Governance systems and processes |  |  |
| Quality and safety systems/initiatives |  |  |
| Outbreak/COVID-19 policies |  |  |
| Policy change process |  |  |
| **POLICIES & PROCEDURES: Training** | | |
| Staff access |  |  |
| Record keeping |  |  |
| **POLICIES & PROCEDURES: Resident deterioration** | | |
| Access to/location of care/deterioration policies/manuals |  |  |
| Management of deterioration roles and responsibilities |  |  |
| Communication with family |  |  |
| ACP policies/records |  |  |
| Hospital transfer |  |  |
| Care post transfer |  |  |
| Decision support tools in use |  |  |
| Use of equipment responding to deterioration:  ECG  Vital signs monitor  Pulse oximeter  Bladder scanner |  |  |
|  | | |
| Internal programs (include promotional and other engagement activities e.g. Dementia Awareness Week) |  |  |
| External programs |  |  |
