# Supplementary material for: From pilot to a multi-site trial: refining the Early Detection of Deterioration in Elderly Residents (EDDIE +) intervention
Source: BMC Geriatr. 2023 Dec 6;23:811. doi: 10.1186/s12877-023-04491-z (PMC10698876; doi:10.1186/s12877-023-04491-z)
Supplement: Supplementary file 3 — Additional file 3. Local RAC Home template. This is the template used by the study team to collect key information about each individual residential aged care home. [file 12877_2023_4491_MOESM3_ESM.docx]

**BASELINE information Home:**

**Date: Completed by:**

Purpose:

- To systematically collect information from each enrolled home to inform local tailoring of all the EDDIE+ activities, including training development and delivery, engagement with staff, resident committees and local primary care providers

| **Information required: RAC HOME**  ***(noting local context and variation to core BC practices/context)*** | **Source** | **Notes** |
| --- | --- | --- |
| **HOME STRUCTURE & CHARACTERISTICS** | | |
| Number of beds (currently occupied and capacity) |  |  |
| Training facilities (room capacity, AV, chairs, tables etc) |  |  |
| Access |  |  |
| Accreditation timings |  |  |
| Recent changes/issues (including outbreaks, occupancy, COVID) |  |  |
| Occupancy |  |  |
| Computer access/connectivity |  |  |
| **PEOPLE: Staff** | | |
| Home staff structure |  |  |
| Local variation to nurse and care staff position descriptions / duty statements |  |  |
| Local variation to roles and responsibilities |  |  |
| Local staff training and education schedule and content |  |  |
| Local new staff induction schedule and content |  |  |
| Structure and processes for nursing and care staff meetings |  |  |
| Local backfill |  |  |
| Staff support networks (mentoring, job coaching) |  |  |
| ACP storage/access/transfer |  |  |
| **PEOPLE: Residents, family and nominated advocates** | | |
| Resident communication mechanisms and frequency |  |  |
| Family and nominated advocate communication mechanisms and frequency |  |  |
| **STAKEHOLDERS: Local primary care providers, other services** | | |
| **Local GP practices:**  Who  Types of engagement  Key personnel |  |  |
| **Local GP practices:**  Who  Types of engagement  Key personnel |  |  |
| **Local GP practices:**  Who  Types of engagement  Key personnel |  |  |
| **Local GP practices:**  Who  Types of engagement  Key personnel |  |  |
| **Other local services**  Who  Types of engagement  Key personnel |  |  |
| **Other local services**  Who  Types of engagement  Key personnel |  |  |
|  |  |  |
| **LOCAL POLICIES & PROCEDURES: Deterioration** | | |
| Management of deterioration roles and responsibilities |  |  |
| Access to/location of care/deterioration policies/manuals |  |  |
| Communication with family |  |  |
| Hospital transfer |  |  |
| Care post transfer |  |  |
| Use of decision support tools (what ones, who uses, where kept) |  |  |
| Use of equipment responding to deterioration:  Vital signs monitor  Bladder scanner | Carron |  |
| **CURRENT PROGRAMS** | | |
| Internal (include promotional and focus activities e.g. Dementia Awareness Week) |  |  |
| External (RaSS, RADAR, GEDI, SPACE, ELDAC, other) |  |  |
| **NOTES** | | |
