# Supplementary material for: From pilot to a multi-site trial: refining the Early Detection of Deterioration in Elderly Residents (EDDIE +) intervention
Source: BMC Geriatr. 2023 Dec 6;23:811. doi: 10.1186/s12877-023-04491-z (PMC10698876; doi:10.1186/s12877-023-04491-z)
Supplement: Supplementary file 4 — Additional file 4. Local RAC Home context template. This is the template used by the study team to assess barriers and facilitators. This i-PARIHS framework was used in its development covering the key domains. [file 12877_2023_4491_MOESM4_ESM.docx]

**BASELINE local context assessment Home: Date:**

Purpose: To systematically identify contextual enablers and barriers to implementation and inform a tailored implementation plan

Based on i-PariHS franework

| **EDDIE+ questions and prompts** | **Enablers** | **Gaps** | **Implementation plan/priorities** |
| --- | --- | --- | --- |
| **1. Characteristics of the innovation (EDDIE+)** | |  |  |
| **Who is directly impacted?** |  |  |  |
| **Underlying knowledge sources & clarity /perception of intervention** |  |  |  |
| **Degree of fit (compatibility or contestability), extent of change required** |  |  |  |
| **Likely boundaries** |  |  |  |
| **Relative advantage** |  |  |  |
| **2. Recipients** | |  |  |
| **Motivation & values and beliefs & consensus** |  |  |  |
| **Skills and knowledge** |  |  |  |
| **Time and resources** |  |  |  |
| **Learning environment** |  |  |  |
| **Existing data sources** |  |  |  |
| **Local opinion leaders** |  |  |  |
| **Collaboration and teamwork** |  |  |  |
| **Power & authority** |  |  |  |
| **Professional boundaries & networks** |  |  |  |
| **3a.       Inner context - local level** | |  |  |
| **Leadership support** |  |  |  |
| **Culture, experience of change, mechanisms for embedding change** |  |  |  |
| **3b.       Inner context - organisational level** | |  |  |
| **(Home) Organisational priorities** |  |  |  |
| **Leadership & senior management support** |  |  |  |
| **Culture / history of innovation and change/systems and processes** |  |  |  |
| **3c. Outer context** | |  |  |
| **Policy drivers & priorities/ incentives & mandates** |  |  |  |
| **Regulatory frameworks** |  |  |  |
| **Environmental (in)stability** |  |  |  |
| **Inter-organisational networks & relationships** |  |  |  |
