# Supplementary figures and images for: From pilot to a multi-site trial: refining the Early Detection of Deterioration in Elderly Residents (EDDIE +) intervention
Source: BMC Geriatr. 2023 Dec 6;23:811. doi: 10.1186/s12877-023-04491-z (PMC10698876; doi:10.1186/s12877-023-04491-z)

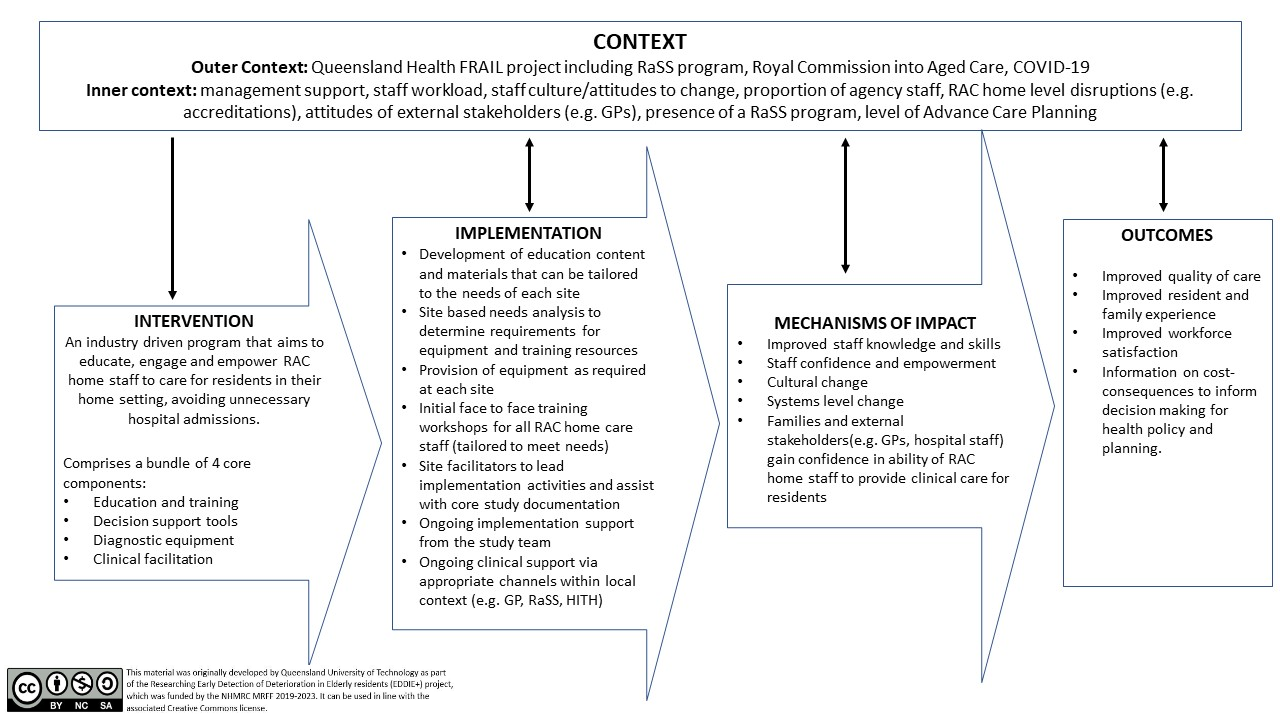

Supplement: Supplementary file 5 — Additional file 5. EDDIE+ Intervention Logic model. This is the program logic model used for the EDDIE+ trial. [file 12877_2023_4491_MOESM5_ESM.png]
