# Supplementary material for: From pilot to a multi-site trial: refining the Early Detection of Deterioration in Elderly Residents (EDDIE +) intervention
Source: BMC Geriatr. 2023 Dec 6;23:811. doi: 10.1186/s12877-023-04491-z (PMC10698876; doi:10.1186/s12877-023-04491-z)
Supplement: Supplementary file 6 — Additional file 6. Fixed and flexible components and degree of flexibility allowed. This table details the fixed and adaptable components of the EDDIE+ intervention and the degree of flexibility that is allowed during the trial phase. [file 12877_2023_4491_MOESM6_ESM.docx]

Additional File6: Fixed and flexible components and degree of flexibility allowed

| **COMPONENT 1: Advanced clinical skills training** | | | |
| --- | --- | --- | --- |
| Elements/key activities | Fixed elements | Flexible elements (to be determined following initial context mapping) | Degree of flexibility (extensive, partial, limited, none) |
| Initial face-to-face training on early identification of deterioration and response, including roles/responsibilities of each staff member. | Initial training will be delivered by a Nurse educator employed as part of the EDDIE+ study.  Training must be attended by all RNs, ENs and PCWs (unless staff are on leave during this period)  Training must cover clinical management practices for specific conditions identified as likely to result in hospitalisation (e.g. UTIs, chest pain, falls, delirium, dehydration, etc).  Training must cover the use of relevant decision support tools and diagnostic equipment.  Training must position EDDIE+ as a research study will a strong evaluation component, and emphasise the focus on generating new evidence to inform decision making. This will include an overview of why the study is needed (i.e. the nature of the problem) and where funding is coming from.  Training must include an element of executive support for the program. | Length, intensity, delivery methods and the depth of content coverage training can be adapted to suit the needs of each site | Partial |
| Educational materials toolkit | A core set of materials will be developed that all sites must cover | Additional materials may be developed to meet needs of each site | Partial |
| **COMPONENT 2: Decision support tools** | | | |
| Elements/key activities | Fixed elements | Flexible elements | Degree of flexibility (extensive, partial, limited, none) |
| Core decision support tool covering clinical decision-making guidelines for managing deterioration across a number of conditions | All sites must implement a core decision support tool that is introduced in initial training and reinforced at staff meetings | - The number and type of conditions covered by the tool may differ depending on the needs at each site. - The form of the tool (e.g. flip chart, online resource, App-based resource) may differ at each site. | Partial |
| Observation chart (track and trigger tool) as hard copy |  | Optional depending on site preferences/requirements | Extensive |
| Communication tool (e.g. Situation, Background, Assessment, Recommendation) |  | Optional depending on site preferences/requirements | Extensive |
| **COMPONENT 3: Diagnostic medical equipment** | | | |
| Elements/key activities | Fixed elements | Flexible elements | Degree of flexibility (extensive, partial, limited, none) |
| Provision of: Bladder scanner, ECG machine, Vital signs monitor, oximeter | Each site will be assessed for their equipment needs and will receive training on equipment as part of the initial face to face training sessions. | The type and amount of equipment will be tailored to meet the needs of each site | Partial |
| **COMPONENT 4: Implementation facilitation and clinical systems support** | | | |
| Elements/key activities | Fixed elements | Flexible elements | Degree of flexibility (extensive, partial, limited, none) |
| **RAC home EDDIE+ Clinical Facilitator** | Each site will have one internal facilitator who is in a clinical leadership position within the RAC home. The EDDIE+ facilitator will have quarantined time (up to 1 day per week) to dedicate to implementation and study activities including stakeholder engagement, record keeping, liaising with EDDIE+ project staff and mentoring/coaching of other care staff. | Time dedicated to facilitation may vary (between 0.1 to 0.2 FTE)  Specific activities may vary depending on context assessment and implementation strategies that are adopted. | Partial |
| **External facilitation and support** | Nurse Educator or Implementation Facilitator will conduct fortnightly meetings/phone calls with each site that has started their intervention phase | Additional facilitation support to be provided by Nurse Educator and Implementation Facilitator (employed as part of the EDDIE+ trial) as required. | Extensive |
| **Facilitator guide** | Facilitator guide to be developed as resource to support EDDIE+ Clinical Facilitators in their role. Will include information on the nature of study team support available throughout the project as well as a set of resources to help guide the facilitation (e.g. templates for documenting engagement activities and/or other data collection requirements). Provided to the EDDIE+ Clinical Facilitator/s at each site |  | Fixed |
| **Ongoing executive/management support** | RAC home leadership to have a presence at the initial training session to reinforce organisational support for the program and confirm their approval for a change in usual practice.  EDDIE+ Clinical Facilitator to coordinate brief but regular communication with RAC home leadership to act as a continuous feedback loop (e.g. fortnightly project updates, standing item at management meetings, etc) | Nature of communication and feedback to suit the context and needs of each site. | Partial |
| **Clinical support channels**: Access to clinical support from medical personnel e.g. hospital in-reach team (e.g. RASS), clinical lead nurses, nurse practitioner, nurse educator, geriatrician, wound specialist) | All sites must establish channels for RNs/ENs/AINs to effectively communicate any concerns regarding patient deterioration and/or need for hospital transfer | Communication channels will be tailored to site context and needs e.g. local hospital avoidance programs, local GP, etc | Partial |
| **Internal staff support networks:** Ongoing internal clinical support from RAC team, senior clinical nurse advisors (SCNAs), clinical facilitators, local GPs. May include elements of ongoing coaching or mentoring. | All sites must establish internal support networks for staff to feel supported in providing the level of care required of the intervention | Nature and form of support networks/channels may be tailored to each site but likely to include access to SCNAs | Partial |
| Alignment of education and decision support materials with organisational policies and procedures to support long term sustainability | Led by the EDDIE+ Clinical Facilitators, all sites to work with study team to align decision support materials with organisational policies and practices, and embed these into business as usual. | Site specific policies and procedures may also need to be aligned with study materials | Partial |
| **GP practice engagement:** GPs to be recognised as key decision makers in RAC home hospital transfers. Initial and ongoing engagement required to build the trust of GPs in RAC home clinical management of residents. | GP engagement activities to occur in all sites | Nature of engagement, and number of practices engaged, to suit needs of individual sites. | Partial |
| **Family engagement:** Families to be recognised as key stakeholders with initial and ongoing engagement activities to occur | Family engagement activities to occur in all sites | Nature of engagement to suit needs of individual sites. | Partial |
