# Supplementary material for: From pilot to a multi-site trial: refining the Early Detection of Deterioration in Elderly Residents (EDDIE +) intervention
Source: BMC Geriatr. 2023 Dec 6;23:811. doi: 10.1186/s12877-023-04491-z (PMC10698876; doi:10.1186/s12877-023-04491-z)
Supplement: Supplementary file 7 — Additional file 7. Changes between pilot intervention and EDDIE+ intervention. This is a table mapping out what was changed between the pilot phase and the final EDDIE+ intervention including the methods and findings of each step. [file 12877_2023_4491_MOESM7_ESM.docx]

| i-PARIHS | Original EDDIE | Process/ Method | Findings | Changes made |
| --- | --- | --- | --- | --- |
| Innovation | Pilot conducted 2013-2016 | Environmental scan | Updated evidence and QHealth clinical pathway available | Inclusion of QHealth Clinical parameters |
|  | Four components:   1. Education and training for all care staff 2. Decision support tools to aid assessment of deterioration 3. Diagnostic equipment 4. Implementation facilitation and clinical systems support | Intervention working group   - Review pilot learnings and program logic - Assess feasibility and sustainability of components - Deconstruct into core and adaptable | Keep the same four components to the program from the pilot, but need to refine/ update them  Clear core and adaptable elements of program for implementation  Decision support tools (Mask-Ed + traffic light) were no longer sustainable due to licensing requirements – need to find alternative  Education/ training content needed to be facilitated and updated  Needed increased focus on PCWs and communication (see recipients) | Employment of EDDIE+ Nurse educator (full time) to update educational content and deliver introductory training to all sites  New decision support tools – point-of care lanyard cards and posters, and assessment tools for nurses |
| Facilitation | Intensive facility-based facilitation by a single nurse/ nurse educator  High engagement with GPs, residents, local hospital | Intervention working group | Need for multi-level facilitation (local and external/project)  Local facilitation must be funded | Employment of internal clinical facilitator role in each site (funded by project – new or existing staff)  External/ project level facilitation for ongoing mentoring and support  Clinical facilitator guide created, and induction session added |
| Recipients | Registered nurses and AINs (personal care role) | Review of educational content (includes review of policies/ procedures with input from Organisation B) | Consider communication style for non-clinical recipients  Increased focus on role of PCWs  Increased focus on communication between PCW and nurses  Need for ongoing training, not just one-off | Create two different training modules and EDDIE+ video  Use “information chunking” for increased retention  Introduction of CUS and Stop and Watch tools  Reinforcement of ISBAR tools  Reminder about importance of feedback loop from nurses back to PCWs  Development of scenarios for use “on the floor” by local clinical facilitator  Encouragement of local level recognition |
| Inner Context | Single site (x2 pilots) – organisation A | Change to 12 sites across Qld - regional and metropolitan with organisation B |  | Introduce organisational and local context assessments |
|  |  | Organisational context assessment | Strong clinical governance structures  Availability of SCNAs  Integrated IT system for resident care information  Current training modules internally on deterioration  No bladder scanner in Qld RACFs - but currently using bladder scanners in another state so able to support implementation in Qld  Current vital signs monitors did not have ECG | Align training content with local policies and procedures with input from Aged Care provider B  Align equipment purchase with contextual requirements and input from SCNA |
|  |  | Local facility level context assessment – NB: Conducted as each site switched over to trial phase not during development phase | Enabled understanding of local needs and dynamics  Hospital avoidance programs led by hospitals not RACFs – support often limited to business hours M-F  Some difficulties with GP accessibility | Able to tailor to suit (within core/ adaptable parameters) |
| Outer Context | No data collected for pilot trial that was available | Environmental scan | Multiple hospital avoidance programs around QLD, as well as some programs for palliative care  Limited inclusion of PCWs in programs and no training materials for PCWs  Royal Commission findings – increased spotlight on aged care sector. Concerns around potential interactions between researchers and residents by Organisation B  Increasing severity of health conditions in RACF cohort (i.e. going to RACF later and sicker) | Organisation B limited the interaction with residents  Organisation B was fearful of being seen as “having an issue” and limited promotion of EDDIE+ and controlled all sharing of information about the research with resident families and stakeholders |
|  | N/A | COVID-19 (impact from early 2020) | Workforce issues (escalation)  Change in priorities for health system  Increased telehealth options  Potential for increased social isolation of residents | Aim for pragmatic implementation  Ensure online options available for training and communication with stakeholders |
